# Hypoglycemic Effect of *Calea urticifolia* (Mill.) DC.

**DOI:** 10.1155/2021/6625009

**Published:** 2021-01-12

**Authors:** Adolfo Andrade-Cetto, Fernanda Espinoza-Hernández, Gerardo Mata-Torres

**Affiliations:** Laboratorio de Etnofarmacología, Facultad de Ciencias, Universidad Nacional Autónoma de México, Avenida Universidad 3000, México 04510, Mexico

## Abstract

The onset of type 2 diabetes (T2D) is a consequence of the progressive loss of adequate *β*-cell insulin secretion, which frequently occurs under a background of insulin resistance. Currently, nearly 13 million Mexicans are living with diabetes. Moreover, due to poor socioeconomic conditions and the cultural idiosyncrasies of the Mexican population, the use of medicinal plants to treat T2D is a common practice in Mexico. In the Mexican state of Hidalgo, we found the traditional use of *Calea urticifolia* (CU) to treat this disease. To treat T2D, people drink an infusion made from the aerial part of the plant throughout the day. With the aim of investigating whether the infusion at a traditional dose produces a hypoglycemic effect in either the fasting or postprandial state, we measured the effect of the infusion in a hyperglycemic animal model (rats administered streptozotocin (STZ) and nicotinamide (NZ)) by conducting a glucose tolerance test and constructing a blood-glucose curve. We then analyzed whether the observed effect was related to the inhibition of glucose absorption in the gut or the inhibition of hepatic glucose output (HGO) *in vivo* and *in vitro*. Furthermore, we confirmed our findings by identifying the potential targets of the infusion via a network pharmacology analysis. Through high-performance liquid chromatography (HPLC) and thin layer chromatography (TLC), we detected a number of compounds in the extract and identified two of them. The plant extract produced a highly significant hypoglycemic effect under fasting conditions and a weak hypoglycemic effect following glucose or sucrose challenge. Although the plant extract blocked only 20% of the alpha-glucosidase enzyme activity *in vitro*, in the pyruvate tolerance test (which measures the liberation of hepatic glucose), it significantly reduced glucose levels. Furthermore, *in vitro*, the extract diminished the activity of the glucose-6-phosphatase complex by 90%. In addition, by conducting TLC, we detected the presence of chlorogenic acid and rutin, which have been reported to block HGO. The results presented here provide evidence of the hypoglycemic effect of the traditionally used *C. urticifolia* extract and demonstrate that this effect is associated with both a reduction in glucose synthesis via gluconeogenesis due to the phytochemical composition of the extract and a slight blockage of glucose absorption in the gut.

## 1. Introduction

Diabetes mellitus is a long-term condition that occurs when there are increased levels of glucose in the blood because the body either cannot produce any or enough insulin or cannot effectively use the insulin it produces.

The onset of type 2 diabetes (T2D) is due to a progressive loss of adequate *β*-cell insulin secretion, frequently under a background of insulin resistance [1]. The International Diabetes Federation (IDF) defines T2D as a condition in which hyperglycemia arises as a result of the inability of the body's cells to fully respond to insulin (insulin resistance). In the period of insulin resistance, this hormone is ineffective, which induces an increase in insulin production. Over time, inadequate production of insulin develops as a result of the failure of pancreatic *β*-cells to keep up with demand [2]. This condition causes serious damage to the heart, blood vessels, eyes, kidneys, and nerves. The IDF reported in 2019 that there was an estimated 463.0 million people living with diabetes worldwide. In their report, Mexico ranked sixth in the world, with 12.8 million patients with diabetes.

T2D can be controlled but not cured. To achieve control from a medical perspective, oral hypoglycemic drugs are used; however, due to the idiosyncrasies of the Mexican culture, medicinal plants are typically used in combination with prescribed treatment [3, 4]. Understanding how medicinal plants work is essential, especially, since they can play a major role in controlling disease, particularly at this time. In July 2020, the Johns Hopkins University (JHU) website reported that Mexico ranked 6^th^ worldwide in the number of COVID-19-infected people and 3^rd^ in global deaths [5], and the Mexican government reported that, at that same time, the second most common comorbidity of people who died due to COVID-19 was diabetes [6]. These observations show that Mexico is far from controlling the general health of diabetic patients and that efforts to improve the glycemic control of diabetic patients are needed.

By consulting T2D patients and healers in the town of Tamala in the Mexican state of Hidalgo, we found the use of *Calea urticifolia* (CU) for the treatment of T2D.

### 1.1. Brief Description of the Plant and Previous Works

*Calea urticifolia* (Mill.) DC. (Asteraceae)—biological form: 0.6–2 m tall, shrub; in general, stems are densely pubescent with patent brown trichomes, especially on top. The leaves are ovate to lanceolate, 3–14 cm long and 1.5–3 cm wide, with an acute to acuminate apex and an acute to rounded base, densely hairy on the underside. Flower and fruit: yellow, corollas, cylindrical achenes, 1.5–3 mm long. The plant was originally distributed from Mexico to Panama. It lives in warm and semiwarm climates, between 22 and 1800 m above sea level. It is associated with disturbed vegetation derived from tropical deciduous, subdeciduous, subevergreen and evergreen forests, and oak and pine forests [7].

Its traditional use has been reported for the treatment of pimples and body irritation, wherein baths are taken in the soaked leaves of the plants. The leaves are applied as a warm poultice to heal sores. It has also been used to combat malaria: juice is obtained by manually crushing the plant and, after dilution with a little water, is drunk in the fasting state. For cough, a decoction of the bark is used. In the event of a biliary effusion, a gargle can be made from an infusion of the plant to provoke vomiting [8]. Some compounds isolated from the roots are thymol, 3-methyl-4-isopropylphenol, and derivatives of dicaffeoylquinic acid [9]. From the leaves, germacranolides; calealactones A, B, and C; 2, 3-epoxycalealactone A [10]; and the flavonoid acacetin [11] have been isolated. Phenolic compounds, such as caffeoylquinic acid derivatives and flavonoid glycosides, have been reported in aqueous extracts from the leaves [12].

The aim of the present study was to investigate whether, at the traditionally used dose and method of consumption, the aerial part of the plant exerts a hypoglycemic effect in fasting and/or postprandial states and, if so, whether this effect is associated with the inhibition of alpha-glucosidases in the gut or the inhibition of hepatic glucose output (HGO).

## 2. Materials and Methods

### 2.1. Ethnobotany and Extract Preparation

Direct interviews were performed with diabetic patients and two herbal specialists who recommend CU for the treatment of T2D. The interviews were free format, with specific questions asked about the recollection, preparation, used doses, and administration form of the plant. With the help of the specialist “Salomón Villegas,” plant material was collected near the town of Tamala in the Mexican state of Hidalgo in 2018 and 2019 in July and August. The plant was identified by the biologist Jorge Rojas (voucher Etnof-238). The extract was prepared in a similar way as the traditionally used tea: 20 g of the dry aerial part of the plant was added to 500 mL of boiling water, and the infusion was stirred for 15 min and then left to rest for 5 min. The infusion was then filtered under vacuum and deep-frozen in a Revco™ freezer. Water was then removed from the infusion by lyophilization in a Labconco™ Freezone 2.5 machine. The extract was stored at 4°C until use.

### 2.2. HPLC and TLC

To determine the phytochemical profile of the tested extract, we performed high-performance liquid chromatography (HPLC) with an Agilent 1260 HPLC instrument equipped with a G1311B quaternary pump, a G1367E autosampler, and an Agilent G1315C UV diode array detector (DAD). HPLC-grade solvents were purchased from JT Baker. Chromatographic profile elaboration was performed using a Phenomenex reversed-phase column (Luna Omega Polar C18, 50 × 2.1 mm i.d., 1.6 *μ*m). Gradient elution was performed with 0.1% aqueous formic acid as solvent A and acetonitrile as solvent B, and the column temperature was maintained at 35°C. System control, data collection, and data processing were accomplished using OpenLAB LC 1260 chromatography software. The working solution of each sample was prepared by dissolving 10.0 mg of the extract in 1 mL of water, and 2 *μ*L of working solution was injected using an autosampler. For UV detection, the wavelength program was set at acquisition wavelength (*λ*) values of 240, 254, 280, 320, and 365 nm. Additionally, thin-layer chromatography (TLC) was performed using known standards as controls. Ten milligrams of dry aqueous extract was weighed and, with the help of a sonicator, dissolved in a solution of 1 mL of water-methanol-acetonitrile (30 : 35 : 35). Then, 15 *μ*L was applied to silica gel Merck F_254_ plates.

### 2.3. Hyperglycemic Animals

Wistar rats weighing 200–250 g were obtained from the Bioterium of the School of Science, UNAM, and acclimated with free access to food (Purina Rodent Laboratory Chow 5001) and water for at least one week in an air-conditioned room (25°C with 55% humidity) on a 12-h light-dark cycle prior to the experiments.

Hyperglycemia was induced as previously described [13, 14]. In brief, rats were fasted overnight and injected intraperitoneally with 150 mg/kg nicotinamide (NA; Sigma N3376). After 15 min, they were injected intravenously with 65 mg/kg streptozotocin (STZ, Sigma S0130) dissolved in acetate buffer (0.1 M, pH 4.5). After 72 h, hyperglycemia was identified based on the presence of polydipsia, polyuria, and measurements of nonfasting plasma glucose levels. Animals that developed one of two glucose thresholds were selected; for experiments 1 and 2, animals with nonfasting glucose levels greater than 180 mg/dL were used, whereas for experiment 3, animals with nonfasting glucose levels greater than 300 mg/dL were selected to obtain 18-h fasted animals with a glucose level of approximately 190 mg/dL. All methods used in this study were approved by the Committee on Academic Ethics and Scientific Responsibility (CEARC) of the School of Science, UNAM (PI_2020_04_001). The study was carried out in accordance with the principles of the Basel Declaration and recommendations from the Committee for the Update of the Guide for the Care and Use of Laboratory Animals [15].

In all experiments, blood samples were obtained from the tail vein. Glucose levels were analyzed every 30 min in duplicate with glucose test strips and Accutrend® Plus glucometers.

### 2.4. Experiment 1: Acute Hypoglycemic Effect (Fasting and Postprandial States)

To determine whether the infusion used at the traditional dose produces a hypoglycemic effect in the fasting state, we performed an acute experiment [16]. In brief, hyperglycemic animals were divided into four groups (1–4) of six rats each: (1) a normal control group in which each rat received 1.5 mL of physiological NaCl solution by gavage (vehicle); (2) a hyperglycemic control group in which each rat received 1.5 mL of physiological NaCl solution; (3) a positive control group in which each rat received the standard oral hypoglycemic agent glibenclamide (Euglucon®) at the dose of 5 mg/kg b.w. in the NaCl vehicle; and (4) an experimental group in which each rat received 41 mg/kg b.w. CU. To determine whether the extract exerts a hypoglycemic effect in the postprandial state, four similar groups were subjected to oral glucose tolerance tests in which glucose (Sigma G7021) was administered 5 min after the following treatments: (5) normal control, physiological NaCl solution; (6) hyperglycemic control, physiological NaCl solution; (7) positive control, 1 mg/kg b.w. repaglinide (Prandin®); and (8) experimental, 41 mg/kg b.w. CU. Glucose, repaglinide, and CU were dissolved in physiological NaCl solution. See the results for the plant dose calculation. In both experimental designs, the rats were monitored for 2 h after treatment.

### 2.5. Experiment 2: Sucrose Tolerance Test (Intestinal Absorption)

To determine whether the extract blocks glucose input in the gut, hyperglycemic animals were classified into 4 groups of eight rats each. All groups received a solution of 3 g/kg b.w. sucrose (Sigma S1888) 5 min after the administration of vehicle, control drug, or extract; the groups were as follows: (1) a normal control group in which each rat received 1.5 mL of physiological NaCl solution (vehicle); (2) a hyperglycemic control group in which each rat received 1.5 mL of physiological NaCl solution; (3) a positive control group in which each rat was treated with acarbose (Glucobay®, 3 mg/kg b.w.); and (4) an experimental group in which each rat received 41 mg/kg b.w. CU. Acarbose and CU were dissolved in physiological NaCl solution. Glucose levels were measured for 1.5 h after treatment.

### 2.6. Experiment 3: Pyruvate Tolerance Test (Hepatic Glucose Output)

To determine if the extract blocks HGO, six groups of six rats each were established [17]: (1) normoglycemic, (2) normoglycemic + pyruvate, (3) hyperglycemic, (4) hyperglycemic + pyruvate, (5) hyperglycemic + pyruvate + 500 mg/kg b.w. metformin, and (6) hyperglycemic + pyruvate + 41 mg/kg b.w. CU. The 18-h-fasted rats were administered vehicle (see above), metformin (Aurax) or extract by gavage; fifteen minutes later, vehicle or sodium pyruvate (2 g/kg b.w., Sigma P5280) was injected intraperitoneally. Sodium pyruvate, metformin, and CU were dissolved in physiological NaCl solution. Glucose levels were monitored for 2 h after treatment.

### 2.7. *In Vitro* Testing

#### 2.7.1. Alpha-Glucosidase Assay

Alpha-glucosidase enzymatic activity was measured as previously described [18] by quantifying the amount of p-nitrophenol released from p-nitrophenyl-alpha-D-glucopyranoside. Each assay volume contained 2 mM p-4-nitrophenol glucopyranoside (p-NPGP, Sigma N1377), phosphate buffer (0.1 M, pH 6.8), alpha-glucosidases from *Saccharomyces cerevisiae* (0.1 U, Sigma G5003) or an intestinal crude extract (Sigma I1630), and 1 mL of the control drug acarbose or the experimental extract at concentrations ranging from 0.2 *μ*g/mL to 1,000 *μ*g/mL. The reaction was monitored for 480 s. Measurements were acquired every 30 s at 405 nm with a BioTek spectrophotometer (model ELx800). Assays were performed in duplicate.

#### 2.7.2. Liver Microsome Isolation

Four overnight-fasted Wistar rats were anesthetized with pentobarbital (6 mg/100 g b.w., i.p.) as previously described [17]. Livers were dissected and homogenized in a 7 mL Dounce tissue grinder to obtain a 20% homogenate buffer (0.25 M sucrose, 1 mM EDTA, 5 mM HEPES, pH 7.4). The homogenate was filtered through nylon mesh and submitted to differential centrifugation 100 000 ×g for 1 h, and the pellets were stored at −40°C until use.

#### 2.7.3. Glucose-6-Phosphatase Assay

A colorimetric assay to assess microsomal glucose-6-phosphatase (G6Pase) inhibition by the extract was performed as described previously [17]. The test consists of the addition of several concentrations of the potential inhibitor from least to greatest concentration in the assay medium, which contains intact rat hepatic microsomes. The reaction was initiated by the addition of substrate and was terminated with the addition of stop solution, which incorporates sodium molybdate and ascorbic acid. A reduced blue phosphomolybdate complex forms due to the presence of the released inorganic phosphate in an amount proportional to the enzymatic activity. In brief, in 100 *μ*L of total assay volume, buffer (40 mM imidazole, 0.25 M sucrose, pH 7), 20 mM glucose-6-phosphate (G6P), microsomes, and chlorogenic acid (control) or CU at different concentrations were added. The reaction was initiated by the addition of G6P, and the volume was incubated at 22°C for 20 min. The reaction was then terminated by the addition of 900 *μ*L of a solution containing 0.42% ammonium molybdate in 1 NH_2_SO_4_, 10% SDS and 10% ascorbic acid. After incubation of the media at 45°C for 20 min, the amount of inorganic phosphate was quantified colorimetrically at 830 nm [19]. Assays were performed in triplicate.

### 2.8. Identification of Potential Targets

To identify some of the potential T2D molecular targets of the main compounds of *C. urticifolia*, a target prediction analysis was performed. The 2D molecular structure and SMILES strings of the main identified compounds were obtained from SciFinder (https://scifinder-n.cas.org/) and DrugBank (https://www.drugbank.ca). The potential molecular targets for the compounds were retrieved from SwissTargetPrediction (http://www.swisstargetprediction.ch), and only activities related to T2D were considered. The functions of the acquired targets were retrieved from the UniProt Knowledgebase (UniProtKB, http://www.uniprot.org/) by restricting the species search to “*Homo sapiens*.” The gene interactions were retrieved from the Comparative Toxicogenomics Database (CTD, http://ctdbase.org/).

### 2.9. Statistical Methods

*In vivo* data are expressed as the mean ± SEM and were assessed for normal distribution with the Kolmogorov–Smirnov test. One-way ANOVA followed by Tukey's *post hoc* analysis was performed to determine the significance of differences between group means. Repeated measures ANOVA followed by Dunnett's *post hoc* analysis was performed to compare each time versus the initial time within the same group. Nonparametric tests were used as appropriate. Area under curve (AUC) data were obtained and expressed as (mg/dL) × min ± SEM. *In vitro* data were expressed as the mean ± SEM, and IC_50_ values were calculated by plotting concentration-response curves to identify the best fitting nonlinear regression model (containing three or four parameters). *p* values less than 0.05 were considered statistically significant.

## 3. Results

### 3.1. Ethnobotany and Plant Yield

As a result of the in-person interviews with the specialists, we determined that the vernacular name of the plant is “Amargoso.” Since the plant has a bitter taste, people believe that if it is consumed as a drink, the bitter flavor counteracts the sweetness of their blood. For this purpose, they prepare an infusion of approximately 20 g of the aerial part of the plant in 500 mL of water, which is drunk between and with meals.

Regarding plant yield, from 20 g of plant, 2.87 g of extract was obtained. The equivalent dose for a one kg rat based on a 70 kg person is 41 mg.

### 3.2. HPLC

**Figure 1:**
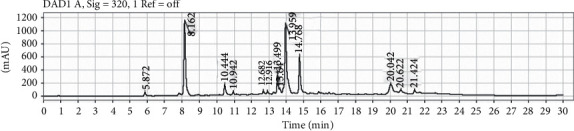
HPLC DAD of the here tested extract.

Figure 1 presents the HPLC-DAD results. Twelve compounds were detected, three of which were present in major proportion. The peaks were found to correspond to previously described caffeoylquinic acid derivatives and flavonoid glycosides [12]. By TLC, the presence of chlorogenic acid and rutin in the active extract was confirmed.

### 3.3. Acute Hypoglycemic Effect (Fasting and Postprandial States)

**Figure 2:**
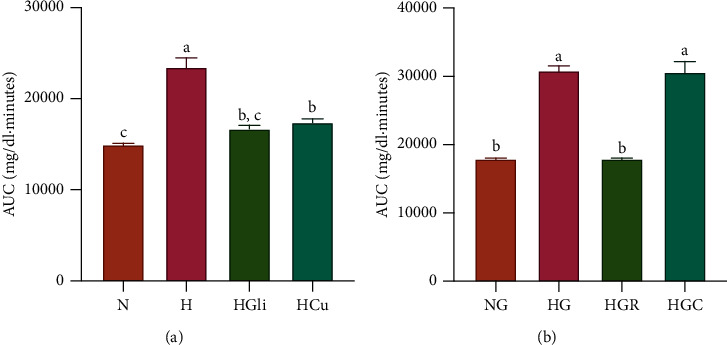
AUC values of *C. urticifolia* hypoglycemic effect curves. (a) Normal curve. (b) Glucose curve. The bars represent the mean ± SEM. Different letters over bars indicate statistically significant differences at *p* < 0.05 (*a* > *b* > *c*). N, normoglycemic group; H, hyperglycemic group; HGli, hyperglycemic + glibenclamide group; HCu, hyperglycemic + *C. urticifolia* group; NG, normoglycemic + glucose group; HG, hyperglycemic + glucose group; HGR, hyperglycemic + glucose + repaglinide group; HGC, hyperglycemic + glucose + *C. urticifolia* group.

In the fasting state, the plant infusion produced a hypoglycemic effect (Table 1). Rats with STZ-NA-induced hyperglycemia (group (2)) presented significantly higher glucose values than the control group (1), whereas the control drug (3) exerted a hypoglycemic effect starting at 30 min when compared with the hyperglycemic group (2) and starting at 60 min when compared with their baseline. On the other hand, the plant infusion (4) produced a hypoglycemic effect at starting at 30 minutes when compared with the control group (2) or its baseline. After a glucose load (Table 2), the control group (5) showed a statistically significant elevation of glucose values, with a maximum increase of 28% observed at 60 min; the values returned to near normal levels after 120 min. In contrast, even though the hyperglycemic control group (6) similarly presented a peak (a 55% increase) in glucose levels at 60 min, the values in this group at 120 min remained much higher than those in the group. Moreover, the control drug repaglinide (7) was able to mitigate the rise in glucose values from 60 to 120 min. The AUC values in Figure 2 show that both CU and glibenclamide had significant hypoglycemic effects (Figure 2(a)). Nevertheless, the infusion of CU could not control glucose levels under glucose challenge (Figure 2(b)).

### 3.4. Sucrose Tolerance Test (Intestinal Absorption)

**Figure 3:**
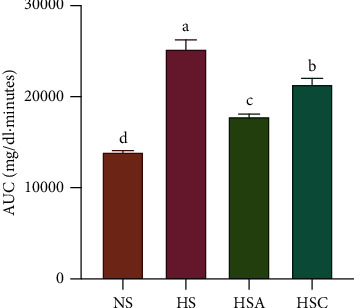
AUC values of *C. urticifolia* sucrose curves. The bars represent the mean ± SEM. Different letters over bars indicate statistically significant differences at *p* < 0.05 (*a* > *b* > *c* > *d*). NS, normoglycemic + sucrose group; HS, hyperglycemic + sucrose group; HSA, hyperglycemic + sucrose + acarbose group; HSC, hyperglycemic + sucrose + *C. urticifolia* group.

After a sucrose load (Table 3), the glucose values of the normal control group had increased by 51% at 30 min; however, they returned to normal after 90 min. However, in the hyperglycemic group, the glucose values had increased by 52% at 60 min and had not returned to basal values after 90 min. The control drug acarbose was able to mitigate the increase in glucose levels from 30 to 90 min, whereas CU was not. Nonetheless, CU was able to return the glucose levels to normal after 90 min. In general, CU had the capacity to produce a significant decrease in glucose levels, as shown by the AUC (Figure 3).

**Figure 4:**
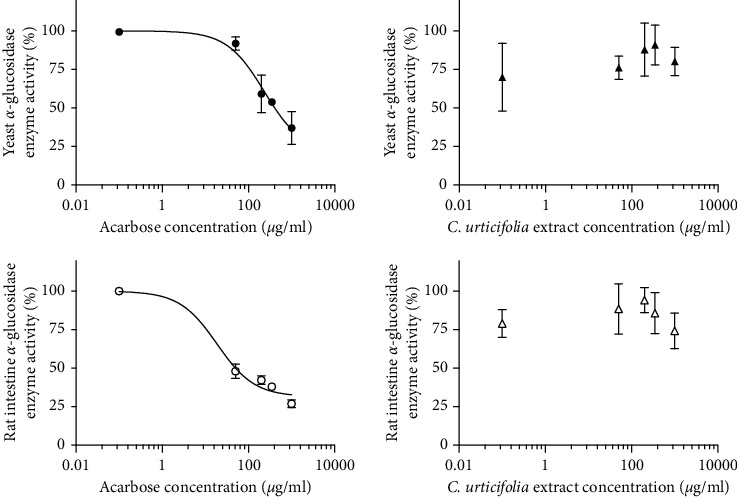
Inhibitory concentration-response curves of acarbose and *C. urticifolia* on *a*-glucosidase activity. Each point represents the mean of two replicates ± SEM.

Although the plant could not fully inhibit the alpha-glucosidases from *S. cerevisiae* or rat intestines, the extract diminished 20% and 26% of enzymes' activity, respectively (Table 4 and Figure 4), which might have contributed to the plant's overall hypoglycemic effect.

### 3.5. Pyruvate Tolerance Test (Hepatic Glucose Output)

**Figure 5:**
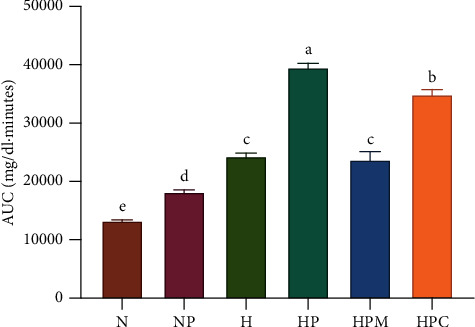
AUC values of *C. urticifolia* pyruvate curves. The bars represent the mean ± SEM. Different letters over bars indicate statistically significant differences at *p* < 0.05 (*a* > *b* > *c* > *d* > *e*). N, normoglycemic group; NP, normoglycemic + pyruvate group; H, hyperglycemic group; HP, hyperglycemic + pyruvate group; HPM, hyperglycemic + pyruvate + metformin group; HPC, hyperglycemic + pyrvate + *C. urticifolia* group.

**Figure 6:**
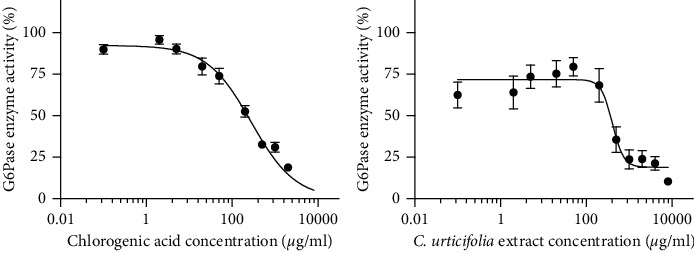
Inhibitory concentration-response curves of chlorogenic acid and *C. urticifolia* on G6Pase system activity. Each point represents the mean of three replicates ± SEM.

When pyruvate was injected into normoglycemic rats, after 60 min, the glucose values were significantly increased by approximately 60% compared with both the values in the control group and the values of the normoglycemic rats at 0 min. After 120 min, the values in the normoglycemic rats had decreased. The values in the hyperglycemic + pyruvate group were increased by 82% after 30 min and remained higher than those in the hyperglycemic control group and their own values at 0 min. In addition, the control drug metformin was able to block the glucose elevation at 30 min compared with the hyperglycemic + pyruvate group and their baseline; however, after 60 min, it also exerted a hypoglycemic effect. In contrast, CU was able to significantly block glucose elevation (Table 5) throughout the monitoring period. Furthermore, the AUC analysis revealed that CU markedly decreased total glucose (Figure 5). In addition, the *in vitro* results showed that CU inhibited 90% of the enzyme activity and yielded an IC_50_ of 406 *μ*g/mL, indicating lower potency of CU than of the chlorogenic acid control (Table 6 and Figure 6).

### 3.6. Target Interactions of the Principal Compounds

Chlorogenic acid acts on two enzymes related to carbohydrate regulation: liver glycogen phosphorylase and aldose reductase. The former enzyme is an important allosteric enzyme in carbohydrate metabolism, and the latter is a key enzyme in the polyol pathway, catalyzing the reduction of glucose to sorbitol during hyperglycemia. However, the function of this compound relies on the electrochemical transporter G6P translocase, which, along with the enzyme G6Pase, forms the complex responsible for the last step of glucose production through glycogenolysis and gluconeogenesis. Hence, the G6P transporter plays a central role in homeostatic regulation of blood glucose levels, and its function is inhibited by chlorogenic acid. Moreover, the top interacting genes are PTGS2, the function of which has been found to be related to hyperglycemia, and COL2A1, the expression of which is related to inflammation.

Rutin interacts with the enzyme aldose reductase and NADPH oxidase 4. The latter may be involved in the regulation of the insulin signaling cascade. The most important genes that interact with this compound are TNF, which is involved in inflammation processes, and CASP3, which is related to a wide variety of processes and diseases, including T2D.

## 4. Discussion

In the practice of Mexican traditional medicine, people commonly use plants to control the hyperglycemia produced by T2D. Patients with diabetes prepare an infusion of *C. urticifolia* that they drink throughout the day, between and with meals. As described above, people believe that the bitter taste of the infusion counteracts the sweetness of their blood.

T2D is a complex pathophysiological disease that involves many metabolic pathways, but its main characteristic is the failure of *β*-cell insulin secretion, frequently in a background of insulin resistance [1]. To date, no animal model with all the characteristics of the disease has been developed; in the present work, we selected the STZ-NA-induced hyperglycemic model, which produces hyperglycemia due to a failure in *β*-cells [13]. Although the model does not properly present insulin resistance, it is useful because glucose levels are moderately high and increase after an injection of pyruvate. In addition, this model responds to the control drugs used here: glibenclamide, repaglinide, metformin, and acarbose.

### 4.1. Hypoglycemic Effect

The results presented here demonstrate that CU produces a hypoglycemic effect that is strong under normal conditions but weak under glucose challenge, and the effect is accompanied by a slight blockade of glucose intestinal absorption. However, the most important effect of the extract is its capacity to block HGO; it is remarkable that this capacity was observed at the traditional dose of 41 mg/kg, which is 10 times lower than the administered dose of the control drug, metformin (500 mg/kg), a pure compound. This observation is consistent with the capacity of the extract to block 90% of the G6Pase activity.

### 4.2. Glucose Absorption in the Gut

Postprandial hyperglycemia is an independent risk factor for cardiovascular disease, stroke, and mortality; it initiates a cascade of prothrombotic and proatherogenic events. It has been shown that a rapid rise in glucose levels increases the activity of low-grade inflammation and thus diabetic complications. Alpha-glucosidase inhibitors lower postprandial blood glucose concentrations, act as competitive inhibitors, and exhibit high affinity for alpha-glucosidases, blocking their enzymatic reactions [20]. In the present study, acarbose blocked 73% of the rat enzyme activity, whereas the CU extract blocked 26% of the activity. Although the effect of CU extract was weaker than that of acarbose, it contributed synergistically to the overall hypoglycemic effect.

### 4.3. Blockade of Hepatic Glucose Output

Endogenous hyperglycemia in T2D is caused by several factors, including increased HGO, triggered by insulin resistance in the fasting state. Poor insulin signaling in the liver cannot efficiently suppress HGO. Overall, hyperglycemia is the sum of two glucose inputs: one from the gastrointestinal tract at the postprandial stage and another from endogenous production. The liver produces approximately 85% of the endogenous glucose, and half of this comes from gluconeogenesis [21]. CU extract acts on both glucose inputs.

In previous works [17, 22], we demonstrated that the pyruvate tolerance test is a good tool to assess the liberation of HGO in the fasting state. G6Pase can control hyperglycemia because it determines the production of glucose released from gluconeogenesis and glycogenolysis [23]; therefore, inhibition of this rate-limiting enzyme is a target to treat hyperglycemia in T2D. The extract inhibits 90% of the enzymatic activity and blocks 55% of the glucose increase under the pyruvate test at 30 min; and, as in the previous case, this effect can contribute in a synergistic way to the total hypoglycemic effect.

### 4.4. Target Interactions

Chlorogenic acid, which was detected in the plant extract, can specifically inhibit the G6Pase T1 translocase system and thus block HGO; it can also raise the phosphorylation level of AMPK in the liver [24]. The other detected compound, rutin, also has hypoglycemic effects, such as decreasing carbohydrate absorption in the small intestine, inhibiting gluconeogenesis, increasing glucose uptake in the liver and muscle tissues, and stimulating insulin secretion from *β*-cells in the pancreas [25]. The target prediction analysis supports these observations: chlorogenic acid blocks the action of G6P translocase, whereas rutin interacts with genes overexpressed in diabetes; furthermore, both compounds can diminish inflammation, which plays an important role in diabetic complications. The compounds detected in the traditional extract can lower glucose levels and reduce complications related to inflammation.

It is remarkable that, as revealed by analysis of the saccharose and glucose curves, the plant does not block glucose input but can lower glucose levels within 90 min of saccharose or glucose challenge. This finding supports the view that the main mechanism of action of CU is the blocking of HGO. Other potential mechanisms not studied here include the enhancement of glucose uptake in peripheral tissues and the augmentation of plasmatic insulin levels.

As we mentioned before, patients with uncontrolled T2D are not only at high risk of diabetic complications but also at higher risk of mortality when infected with agents such as the new SARS-CoV-2 virus. Considering the high mortality rate in Mexico due to COVID-19 and many of the COVID-19-related deaths are associated with a diabetic state, the use of plants such as CU in combination with prescription medications could provide better glycemic control for T2D patients.

In conclusion, we provide evidence that CU exerts a hypoglycemic effect that is associated with the method of consumption and arises through two mechanisms: when the plant is consumed with meals, it can partially block glucose absorption, and when it is consumed between meals, it blocks HGO, contributing to the suppression of the main factors that cause hyperglycemia. Furthermore, we provide evidence of the mechanistic effects of CU at the traditionally used dose, the main action of which is the blockage of HGO, specifically G6Pase, due to its chlorogenic acid and rutin contents.

## Figures and Tables

**Table 1:** Blood glucose levels under normal curve.

Group/time (min)	Glucose levels in the normal curve (mg/dL)				
	T0	T30	T60	T90	T120
1. Normoglycemic control	120 ± 3^b^	125 ± 3^b^	125 ± 3^b^	128 ± 4^b^	121 ± ^b^
	100%	105%	105%	107%	102%
2. Hyperglycemic control	188 ± 3	204 ± 9	201 ± 8	192 ± 11	183 ± 13
	100%	109%	107%	102%	97%
3. Hyperglycemic + glibenclamide, 5 mg/kg	182 ± 4	158 ± 6^b^	134 ± 5^a,b^	118 ± 4^a,b^	111 ± 4^a,b^
	100%	88%	74%	65%	61%
4. *Calea urticifolia* (Mill.) DC., 41 mg/kg	182 ± 5	151 ± 6^a,b^	141 ± 5^a,b^	135 ± 6^a,b^	123 ± 6^a,b^
	100%	83%	78%	75%	68%

The values represent the mean ± SEM; in the same row, *a* indicates statistically significant differences compared with time 0; in the same column, *b* indicates statistically significant differences compared with the hyperglycemic control group; *p* < 0.05, *n* = 6.

**Table 2:** Blood glucose levels under glucose curve.

Group/time (min)	Glucose levels in the glucose curve (mg/dL)				
	T0	T30	T60	T90	T120
5. Normoglycemic control	124 ± 1^b^	158 ± 4^a,b^	159 ± 3^a,b^	149 ± 2^a,b^	139 ± 4^a,b^
	100%	127%	128%	120%	112%
6. Hyperglycemic control	182 ± 3	265 ± 12^a^	282 ± 7^a^	275 ± 9^a^	230 ± 7^a^
	100%	146%	155%	151%	126%
7. Hyperglycemic + repaglinide, 1 mg/kg	175 ± 4	222 ± 8^a,b^	171 ± 7^b^	142 ± 4^a,b^	127 ± 2^a,b^
	100%	127%	98%	81%	73%
8. *Calea urticifolia* (Mill.) DC., 41 mg/kg	180 ± 4	273 ± 15^a^	294 ± 20^a^	256 ± 14^a^	217 ± 9^a^
	100%	152%	163%	142%	121%

The values represent the mean ± SEM; in the same row, *a* indicates statistically significant differences compared with time 0; in the same column, *b* indicates statistically significant differences compared with the hyperglycemic control group; *p* < 0.05, *n* = 6.

**Table 3:** Blood glucose levels under sucrose curve.

Group/time (min)	Glucose levels in the sucrose curve (mg/dL)			
	T0	T30	T60	T90
Normoglycemic control	123 ± 2^b^	185 ± 2^a,b^	155 ± 3^a,b^	125 ± 3^b^
	100%	151%	126%	102%
Hyperglycemic control	200 ± 6	303 ± 11^a^	305 ± 18^a^	267 ± 17^a^
	100%	152%	153%	134%
Hyperglycemic + acarbose, 3 mg/kg	196 ± 3	202 ± 3^b^	197 ± 4^b^	195 ± 3^b^
	100%	103%	101%	100%
*Calea urticifolia* (Mill.) DC., 41 mg/kg	186 ± 3	275 ± 6^a,b^	252 ± 13^a,b^	184 ± 8^b^
	100%	148%	136%	99%

The values represent the mean ± SEM. In the same row, *a* indicates statistically significant differences compared with time 0. In the same column, *b* indicates statistically significant differences compared with the hyperglycemic control group. *p* ≤ 0.05, *n* = 8.

**Table 4:** *In vitro* results with *α*-glucosidase enzymes.

Inhibitor	*S. cerevisiae*		Rat intestine	
	*α*-Glucosidase enzymes		*α*-Glucosidase enzymes	
	Inhibition percentage	IC_50_ (*μ*g/mL)	Inhibition percentage	IC_50_ (*μ*g/mL)
Acarbose (control)	64	231.9	73	18.6
*C. urticifolia* extract	20	—	26	—

**Table 5:** Blood glucose levels under pyruvate curve.

Group/time (min)	Glucose levels in the pyruvate curve (mg/dL)				
	T0	T30	T60	T90	T120
Normoglycemic control	111 ± 4	118 ± 3	109 ± 2	105 ± 2	110 ± 4
	100%	106%	98%	95%	99%
Normoglycemic control + pyruvate	106 ± 4	169 ± 4^a,b^	168 ± 7^a,b^	151 ± 9^a,b^	135 ± 9^a^
	100%	159%	158%	142%	127%
Hyperglycemic control	190 ± 5	211 ± 6^a,b,c^	204 ± 7^b,c^	202 ± 10^b,c^	199 ± 11^b,c^
	100%	111%	107%	106%	105%
Hyperglycemic control + pyruvate	196 ± 4	359 ± 12a	353 ± 13a	357 ± 19a	351 ± 17a
	100%	182%	180%	182%	179%
Hyperglycemic + pyruvate + metformin, 500 mg/kg	211 ± 9	247 ± 16^a,c^	193 ± 21^c^	170 ± 24^c^	152 ± 26^c^
	100%	117%	91%	81%	72%
*Calea urticifolia* (Mill.) DC., 41 mg/kg	195 ± 9	283 ± 16^a,c^	302 ± 15^a,c^	320 ± 13^a,c^	320 ± 16^a,c^
	100%	145%	155%	164%	164%

The values represent the mean ± SEM; in the same row, *a* indicates statistically significant differences compared with time 0; in the same column, *b* indicates statistically significant differences compared with the normoglycemic control group and *c* indicates statistically significant differences compared with the hyperglycemic + pyruvate group; *p* ≤ 0.05, *n* = 6.

**Table 6:** *In vitro* results with glucose-6-phosphatase enzyme complex.

Inhibitor	Inhibition percentage	IC_50_ (*μ*g/mL)
Chlorogenic acid (control)	100	278
*C. urticifolia* extract	90	406

## Data Availability

The data used to support the findings of this study are included within the article.
